# Feasibility of promoting physical activity using mHEALTH technology in rural women: the step-2-it study

**DOI:** 10.1186/s12905-021-01561-5

**Published:** 2021-12-16

**Authors:** Manorama M. Khare, Kristine Zimmermann, Rebecca Lyons, Cara Locklin, Ben S. Gerber

**Affiliations:** 1 Director, Division of Health Research and Evaluation, Department of Family and Community Medicine, University of Illinois College of Medicine Rockford, 1601 Parkview Ave, Rockford, IL 61107 USA; 2 Division of Health Research and Evaluation, Department of Family and Community Medicine, University of Illinois College of Medicine Rockford, Rockford, USA; 3 Community Health Sciences Division, School of Public Health, University of Illinois Chicago, Chicago, USA; 4 Director of Public Health Emergency Preparedness, Winnebago County Health Department, Rockford, USA; 5 Center for Research on Women and Gender, University of Illinois at Chicago, Chicago, USA; 6 Division of Health Informatics and Implementation Science, University of Massachusetts Chan Medical School, Worcester, USA

**Keywords:** Rural Women’s Health, Physical activity, mHealth, Physical activity trackers, Rural health

## Abstract

**Background:**

Rural women are more likely to be obese and have a higher risk for chronic disease than their non-rural counterparts. Inadequate physical activity (PA) at least in part contributes to this increased risk. Rural women face personal, social and environmental barriers to PA engagement. Interventions promoting walking among rural women have demonstrated success; however, few of these studies use text messaging to promote PA.

**Methods:**

Step-2-It was a pilot study to assess the feasibility, acceptability, and effectiveness of text-messaging combined with a pedometer to promote PA, specifically walking among English-speaking women, aged 40 and older, living in a rural, northwest Illinois county. Enrolled participants completed baseline assessments, received pedometers and two types of automated text messages: motivational messages to encourage walking, and accountability messages to report pedometer steps. Participants engaged in 3, 6, 9, and 12-week follow-ups to download pedometer data, and completed post-intervention assessments at 12 weeks.

**Results:**

Of the 44 enrolled participants, 35 participants (79.5%) completed the intervention. Among completers, the proportion meeting PA guidelines increased from 31.4% (11/35) at baseline to 48.6% (17/35) at post-intervention, those with no PA decreased from 20% (7/35) to 17.1% (6/35). During weeks 1-12, when participants received motivational text messages, average participant daily step count was 5926 ± 3590, and remained stable during the intervention. Pedometer readings were highly correlated with self-reported steps (r = 0.9703; *p* < 0.001).

**Conclusion:**

Step-2-It was a feasible and acceptable walking intervention for older rural women. Technology, including text messaging, should be investigated further as an enhancement to interventions for rural women.

*Trial Registration on Clinicaltrials.gov*: NCT04812756, registered on March 22, 2021

## Background

Rural women are significantly more likely to be obese than non-rural women [1], which is associated at least in part to inadequate physical activity (PA) [2–6]_._ It is well established that physical activity (PA) can lower the risk for heart disease, stroke, type 2 diabetes, and certain cancers [2] and that *physical in*activity is the fourth leading risk factor for mortality worldwide [4]. Given these associations between PA engagement and mortality as well as disease risk, and that PA levels typically decrease with age [10], interventions to encourage PA among rural women who are middle-aged and older are essential. Rural women report personal barriers to PA engagement including the lack of time and injury, as well as barriers in their physical and social environments, including limited access to exercise facilities and lack of social support [7–9].

Walking is an affordable PA option that in part addresses the barriers to PA engagement and is associated with a low injury risk [2]. Previous interventions to encourage walking, including those implemented in rural communities, have achieved success in increasing PA, weight loss and improving risk factors for multiple chronic diseases [11–17].

Mobile health (mHealth) technology and PA trackers are two technologies that can be used to promote walking in rural women.

Mobile health, or mHealth, is the application of wireless devices to support medical or public health practices [18]. Text-messaging is one mHealth strategy that has the ability to reach a large number of people at a relatively low cost [19]. Cell phone use in the US is nearly universal, 97% of urban adults and 95% of rural adults are cell-phone users [19]. mHealth interventions using text messaging have been used to address a variety of health concerns [20–26], including PA promotion [27–29]. However, few studies have examined the use of mobile health technologies to promote PA in rural women [30], and to our knowledge, no such studies have focused on midlife and older, rural women. Mobile health or mHealth when used to promote walking in rural women can broaden the reach of these interventions and gives the ability to communicate with women in real-time.

Physical activity trackers and wearable devices are easily available and affordable and there is evidence to show that their use is helpful in promoting increasing physical activity. In the America on the Move study, adults who were using a pedometer accumulated significantly more steps than those who were not [31]. This finding suggests that using physical activity tacking devices such as pedometers might motivate individuals to increase their physical activity and is supported by a review that shows that pedometer-based walking programs increased participants’ activity levels by 2,183 steps per day [15]. A systematic review [4, 32] of 14 mobile health interventions with physical activity as an outcome showed that 7 of the 14 showed significant positive benefits on self-reported physical activity outcomes.

Step-2-It was a pilot study designed to evaluate the feasibility, specifically acceptability and preliminary effectiveness [33], of text-messaging combined with a pedometer to promote PA, particularly walking, among midlife and older women, residing in a rural Illinois county. The Step-2-It pilot study assessed the of feasibility of text-messaging combined with a pedometer to promote PA among rural midlife and older women. The feasibility assessment included acceptability of Step-2-It, as measured by participant enrollment, engagement with text messages and pedometers, retention in the program, and program satisfaction, as well as preliminary effectiveness in improving PA outcomes [33].

## Methods

### Study design

This prospective, one-group, pre-post study targeted women ages 40 and older living in Stephenson County, IL.

### Setting

At the time of this study, Stephenson County, in rural, northwest Illinois, had a population of 47,315 [34]. It is considered “nonmetropolitan” based on the Rural Urban Continuum Codes [35]. The county is predominantly white, non-Hispanic (87%), with a median age of 43.7 years [34]. Women living in Stephenson County have a high prevalence of chronic disease risk factors. In 2013, 36% reported having high blood pressure, 37% had high cholesterol, 38% were overweight, and 22% were obese [36]. Additionally, 46% did not meet weekly PA standards and an additional 11% were inactive [36].

This study was conducted between August 2014-January 2015 in collaboration with the Stephenson County Health Department (SCHD). This study was approved by the University of Illinois at Rockford Institutional Review Board.

### Inclusion and exclusion criteria

Inclusion criteria were English-speaking, women aged 40 and older, residing in Stephenson County, owned a cell phone with texting capability, and an unlimited texting plan.

Exclusion Criteria were a self-reported diagnosis of bronchitis, pneumonia, or severe asthma, or those being treated for a severe health conditions.

### Enrollment

Flyers advertising the study were placed in the grocery stores and the health department, and interested participants called the lead researcher who assessed eligibility using a checklist. Eligible women were invited to one of five enrollment meetings at SCHD. At least two follow-up calls were made to interested women who did not attend their scheduled enrollment meeting to invite them to the next meeting. A total of 56 women were screened and all met the study eligibility criteria; 44 women (78.6%) came for the enrollment appointment and were enrolled in the study. At the enrollment meetings, participants provided written consent, received a pedometer (Omron HJ-720ITC) and were trained on how to use it. In addition, they completed a series of baseline instruments detailed below and were provided educational materials on cardiovascular disease risk reduction through PA and healthy eating.

### Intervention

There were two components to the 13-week, Step-2-It intervention: (i) participants used the pedometer to track and report their steps via text message daily; and (ii) participants received an informational or motivational text message daily.

*Pedometer/Step Reporting:* Starting in week 0, participants self-reported daily step counts via text message, and continued to do so for the duration of the 13-week intervention. Participants received a daily text reminder to report their steps. Those who did not report steps for two days in a row received a reminder phone call. Every three weeks, participants returned to SCHD to have their pedometer data downloaded. Participants received a $5 incentive for each download, for a total of $20 over 13 weeks.

*Text messaging:* Text messages were sent using mytapp, an online application that allowed for scheduling individual and recurring messages via a cloud service, Twilio. Text messages used for this study were limited to 160 characters and participants could choose their preferred time of day to receive texts. In week 0, the only message participants received were to remind them to report their steps. From week 1-12, in addition to the reminder to report steps, participants received one informational or motivational text message per day (7 messages/week). All participants received the same text messages each day.

Motivational messages were based on social cognitive theory [37] with the intention of increasing participant self-efficacy to engage in PA. These messages were adapted from a database of messages from a previous study to increase PA among African American breast cancer survivors [38]. Adaptations were made to the messages based on two focus groups conducted with 20 women in the target population prior to this study (unpublished study). Sample motivational messages included, *“Nothing is impossible. The word itself says “I’m Possible!”and “Always focus on how far you have come, rather than how far you have left to go.”*

Informational messages included local, PA-related events and resources, such as walks and low-cost walking options. Sample information messages included, *“Walk for a cause – sign up for charity walks”* and *“Find a friend to walk with.”* In addition, there were also messages that reminded women to walk. These messages included, *“Check out what is going on outside, go for a walk”*, “*Don’t just think about it, actually go for a walk*”, and *“Take a quick walking break.”*

### Data collection and measures

Data collected for this study along with the time-points at which they were collected are described below.

#### Participant characteristics (collected at baseline)

Participant demographic information including age, race, education, marital status, employment, and household income; and health status information including self-reported previous diagnosis of diabetes, hypertension, high blood cholesterol, and other heart health diagnoses, medication use for chronic conditions, and tobacco use.

#### Physical activity questionnaire (collected at baseline and post-intervention)

Self-reported PA was assessed using seven questions from the 2005 Behavioral Risk Factor Surveillance System (BRFSS) Physical Activity questionnaire [39]. These data were used to determine whether participants met the recommended aerobic PA levels. Questionnaire reliability and validity have been established previously [40].

#### Physical activity pedometer readings (collected at weeks 3,6,9, and 12) and self-reported steps (collected daily)

The primary outcome measure was number of steps. The Omron HJ-720ITC pedometer, validated in previous studies [41–43], was used for an objective measure of steps. Data was downloaded from the pedometers every three weeks. Participants also self-reported daily steps via text message.

#### Body weight (collected at baseline and postintervention)

Weight was measured using a calibrated scale at SCHD.

#### Intervention satisfaction (collected at post-intervention)

Was assessed using a survey that included questions on overall perceptions about Step-2-It, number of messages received, preferred message types, perceptions about the effectiveness of text messages in promoting health and PA and plans to continue PA after participating in Step-2-It. Participants were also asked about barriers to reporting steps. Participants who completed this survey received a $20 incentive.

### Data analysis

Participants who completed the post-intervention assessment (35/44) were included in the analysis. Descriptive statistics were used to characterize the study population and assess post-intervention satisfaction. Baseline to post-intervention comparisons for body weight were conducted using the paired categorical Wilcoxon Sign rank test. Comparisons of BRFSS PA level was conducted using the Chi-square test. Self-reported step data were compared to downloaded step data and a Pearson correlation was calculated. Correlation testing for self-reported steps versus pedometer-recorded steps excluded observations where only one variable was present. For analyzing mean steps, missing pedometer data was filled in with self-reported step data. Mean steps by week of intervention was calculated and a box plot of mean daily steps by intervention week was produced. We compared step counts at week 0 and week 12 using a matched pair comparison.

## Results

### Participant demographics

Table 1 presents the demographic characteristics of the participants included in the analysis. The mean age of the participants was 53.4 ± 8.1 years (range: 43-74). The mean weight of the participants was 195.8 ± 40.8 lbs. A significant proportion of the participants (37%) reported at least one chronic disease condition, 40% reported 2 chronic disease conditions and 5.7% reported 3 chronic disease conditions. At baseline, majority of participants (48.6%, 17/35) did not meet moderate PA guidelines and 20% (7/35) reported no moderate PA. Eight of 44 participants (18.2%) were lost to follow-up. These 8 participants stopped reporting steps and/or attending meetings for download of step data. One additional participant did not complete the post-intervention PA assessment. A comparison of completers and dropouts showed no significant differences in the proportion of participants who met the moderate activity PA guidelines, and in baseline demographic and health history variables (data not shown).

**Table 1:** Baseline characteristics of step-2-It participants

Characteristics	Enrolled participants (n=35) n (%)
Mean age (mean ± sd)	53.4 ± 8.1
Race/ethnicity	
White, non-Hispanic	27 (77.1)
Other	8 (22.9)
Education	
Some high school/High school/GED	6 (17.1)
Some college	14 (40.0)
Associate’s degree	5 (14.3)
Bachelor’s degree	10 (28.6)
Marital status	
Married, Unmarried living with a partner	20 (57.1)
Unmarried, Divorced, Separated, Widowed	15 (42.9)
Employment status	
Employed for wages	24 (68.6)
Not employed for wages	8 (22.9)
Missing	3 (8.6)
Household income	
Less than $15,000	4 (11.4)
$15,001-$35,000	15 (42.9)
$35,001or more	16 (45.7)
Chronic disease indicators^a^	
Type II Diabetes	3 (6.8)
Pre-diabetes	6 (17.1)
High blood pressure	10 (28.6)
Pre-hypertension	3 (8.6)
High cholesterol	16 (45.7)
Smoke daily	4 (11.4)
Mean body weight (lbs) (mean ± sd)	195.8 ± 40.8
BRFSS physical activity measures	
No moderate physical activity	7 (20.0)
Does not meet moderate intensity activity standards	17 (48.6)
Meets moderate intensity activity standards	11 (31.4)

^*a*^Categories are not mutually exclusive

### Physical activity as measured by the BRFSS tool

From pre-intervention to post-intervention, the percentage of participants with no moderate intensity PA fell from 20.0% (7/35) at baseline to 17.1% (6/35) after the intervention. The number of individuals meeting moderate PA guidelines increased from 31.4% (11/35) to 48.6% (17/35) presented in Table 2 (Overall p-value for Chi-square 0.329).

**Table 2:** Change in self-reported moderate physical activity levels among Step-2-It participants (n = 35) from baseline to post-intervention

	Baseline n (%)	Post intervention n (%)	
Meets (≥30 mins, ≥5 days/wk)	11 (31.4%)	17 (48.6%)	
Less than recommended	17 (48.6%)	12 (34.3%)	Χ^2^ = 2.225, p = 0.329, α = 0.05
No activity	7 (20.0%)	6 (17.1%)	

Physical activity levels assessed by the BRFSS Physical Activity Questionnaire

### Step data

**Fig. 1:**
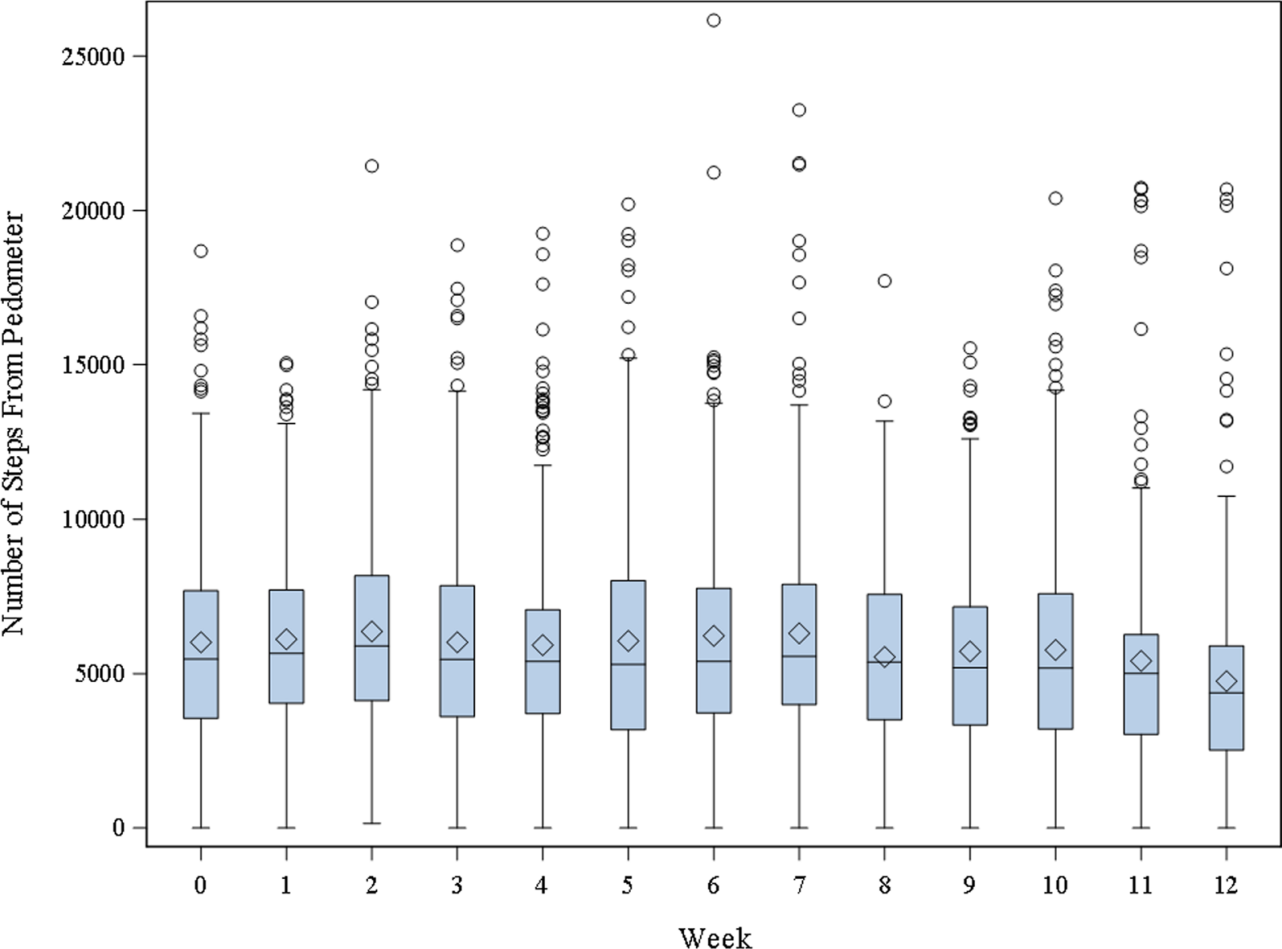
Mean daily pedometer step counts by week for Step-2-It participants (n=35)

The mean daily step count in week 0 (baseline week) was 6,090 ± 3,493, and the mean for weeks 1-12 (the intervention period) was 5926 ± 3590. The mean step count in week 12 was 4,782 ± 3,328. Figure 1 shows the mean daily steps per week for all participants. There was a high positive correlation between the self-reported steps and the step data downloaded from the pedometer (r = 0.9713, n = 2812, *p* < 0.001).

### Change in body weight

The mean weight change from baseline to the end of the intervention was -0.84 ± 7.31 lbs

(n = 35, range: -29.2 to 8.9 lbs, p=0.976).

### Satisfaction with program

All participants who completed the post-intervention questionnaire (n=35) seeking feedback on the program reported being satisfied with their experience in Step-2-It and said that it was easy for them to use their pedometers and report their daily steps via text message. Most participants (88.5%) reported that receiving text messages helped them increase the amount of physical activity or walking they did. Most participants (82.9%, 29/35) indicated they would continue to use their pedometers after Step-2-It was over, 5 (14.3%) indicated that they were uncertain and only one participant did not plan to use her pedometer.

### Types of messages

When asked about which types of messages were their favorite, 57.1% mentioned motivational messages, 51.4% mentioned messages that reminded them to walk, and 65.7% mentioned informational messages about walking.

### Effect of daily step reporting on physical activity

When asked about the daily text messages reminding participants to report their steps, participants indicated daily reporting: helped them walk more (51.4%), made them think about physical activity more (80.0%), helped them make walking a part of their daily routine (45.7%), and motivated them to walk more (40.0%). Two participants (5.6%) reported that daily text messages did not affect their motivation, attitudes, or behaviors related to physical activity. Post-intervention survey respondents also indicated that sending their daily steps via text messages played a role in their PA.

The most common reasons given for not reporting steps were forgetting to respond, not having time to respond, losing, or misplacing the pedometer, or a pedometer malfunction. Two people reported lack of cell phone reception as a barrier.

Suggestions provided by participants for future programs included more facts on walking, customized texts related to the user’s individual goals, and a competition between participants to create a challenge and motivate them.

## Discussion

mHealth strategies have commonly been used for health promotion, chronic disease management, patient communication and treatment adherence. These strategies have also been used with urban populations and in younger age groups. This study demonstrates the feasibility and acceptability of using mHealth—specifically text messaging combined with physical activity trackers—to promote PA in rural women ages midlife and older. Given the high rates of obesity and physical inactivity among aging rural women [4–6, 10], feasible and acceptable strategies for engaging this population in PA are essential.

This study used motivational and informational messages to encourage PA engagement, which has demonstrated significant benefits in self-reported physical activity outcomes in previous studies [44]. While text messaging alone may be insufficient for supporting changes in PA behavior [45], our study used bi-directional messaging by sending participants a daily message to report their steps. This strategy potentially encouraged continued engagement in the program and may have increased participant accountability and motivation to walk more regularly. Similar results have been reported in the one study we found conducted with rural residents [30]. While wireless PA trackers have the advantage of allowing interventionists and researchers to track steps in real time without directly engaging participants [45], the current study suggests regular accountability via one-on-one engagement may be important for older rural women because it provides support to sustain the behavior.

Notably, text messaging has the potential to achieve a broad reach in rural communities at a relatively low cost [32], and may be particularly useful in rural communities that have limited broadband connectivity. According to the FCC’s First Communications Market Report, as of 2017, 24% of Americans in rural areas lacked coverage as compared to only 1.5% of Americans in urban areas [46]. Given the gaps in broadband access as well as rural PA opportunities [46], future research should investigate the effectiveness of tailored messages for older, rural woman, such as individualized goal setting based on the prior week’s steps, providing PA tips when weather does not support outdoor PA, and helping women resume activity after an illness.

While our participants overall did not demonstrate an increase in daily steps, we believe this was because we provided pedometers at the time of enrollment. The average number of steps in the baseline week was higher than that reported in other studies [4]. It is likely that providing the pedometer to collect baseline steps worked as an intervention itself, and motivated women to increase their PA. Hence, our baseline measure may have been greater than pre-pedometer levels, which could be a reason for not detecting a difference in mean steps pre- and post-intervention. Despite the lack of change in steps, the proportion of participants with no moderate PA decreased and the proportion that met moderate PA guidelines increased. This finding supports our belief that pedometers acted as an intervention, and that participants increased their moderate PA because of this intervention. We compared the self-reported steps (a subjective measure) with pedometer step data (an objective measure) to establish that both measures were equally reliable.

This study is limited in that it was a pilot study with a small sample size and lack of a comparison group. Additionally, the study was unable to capture a true baseline for PA. Despite these limitations, our study shows that texting is a feasible and acceptable way to provide health promotion messages to a geographically isolated, hard-to-reach population of rural women.

## Conclusions

We were able to demonstrate that providing pedometers along with motivational and informational text messages increased walking in rural women. Additionally, texting is an effective way to reach residents in rural communities where broadband accessibility is limited. Future studies using comparison groups and an objective PA measure, such as accelerometers, are warranted.

## Data Availability

The datasets for this study are available from the corresponding author on reasonable request.

## Abbreviations

PA: Physical Activity
mHealth: Mobile health
SCHD: Stephenson County Health Department
BRFSS: Behavioral Risk Factor Surveillance Survey
